# Determining frequencies of fungicide resistance in *Leptosphaeria maculans* that render demethylation inhibitor fungicides ineffective in the field

**DOI:** 10.1002/ps.70780

**Published:** 2026-03-31

**Authors:** Alec J McCallum, Alexander Idnurm, Stephen J Marcroft, Andrew H Ware, Elizabeth M Sheedy, Angela P Van de Wouw

**Affiliations:** ^1^ School of BioSciences, University of Melbourne Melbourne Australia; ^2^ Marcroft Grains Pathology Horsham Australia; ^3^ EPAG Research Port Lincoln Australia

**Keywords:** blackleg, canola, DMI, fungicide efficacy, fungicide resistance

## Abstract

**BACKGROUND:**

Fungicide resistance is a significant issue affecting disease control in crops worldwide. Resistance to demethylation inhibitor (DMI, Fungicide Resistance Action Committee Group 3) fungicides has evolved in *Leptosphaeria maculans* and is widespread at high levels across Australia. Molecular tools have recently been developed for the *ERG11* gene to monitor and quantify DMI resistance in *L. maculans* populations. Here, these tools were used to: (i) determine the frequency of resistance in a population needed for the fungicide to become ineffective in controlling disease, (ii) test current levels of resistance in Australian populations, and (iii) explore potential fitness penalties in strains with resistance.

**RESULTS:**

Glasshouse experiments demonstrate that the frequency of fungicide resistance needed to cause a loss of fungicide efficacy is between 1% and 10%. The frequency of resistance can also increase by up to 34.4% following just one application, but in the absence of a fungicide there is limited decrease in the frequency of fungicide resistance, indicating a lack or limited fitness penalty in *ERG11* mutants. In the field, the frequency of DMI resistance in *L. maculans* populations varied from 1.8% to 30.0%, with varying levels of field failure observed in all but the lowest frequency, but there was a poor correlation between DMI resistance frequency and DMI fungicide efficacy.

**CONCLUSION:**

A loss of control of blackleg disease with DMI fungicides can occur when 1–10% of the population is DMI fungicide resistant, with all Australian *L. maculans* field populations tested being within or above this range. Therefore, for many areas in Australia, applications of DMI fungicides may not provide adequate or expected levels of disease control. Fungicide resistance is not the only determinant of fungicide efficacy and other factors such as infection timing, disease pressure and climate may also contribute. To maintain disease control and high yields, farmers in these areas may need to consider using non‐chemical control methods or use other fungicide modes of actions. © 2026 The Author(s). *Pest Management Science* published by John Wiley & Sons Ltd on behalf of Society of Chemical Industry.

## Graphical abstract

As little as 1–10% of the population is needed to be resistant for demethylation inhibitor fungicides to be rendered ineffective. Following a single round of selection, the frequency increases by more than 15% with limited fitness penalty detected when no selection is present.

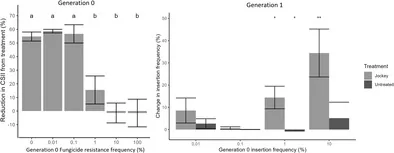

## 1 INTRODUCTION

The evolution of fungicide resistance is an issue for disease control in crops worldwide.^1^ Annual crop yield loss from pathogens and pests is significant and the use of fungicides is an important tool in controlling plant diseases.^2^ The failure of fungicides to control disease may occur for several reasons such as incorrect timing, low dosage, poor application method or fungicide resistance.^3^ Fungicide resistance is caused by mutations that allow a pathogen to survive in the presence of a fungicide and continued use of the fungicide results in a build‐up of resistance and eventual loss of efficacy of that chemical.^3^ These mutations may occur in the coding or promoter region of the target gene or in other genes such as efflux transporters.^4^ ‘Field failure’, also referred to as practical resistance, of a fungicide occurs when the frequency of resistance mutations in a population reaches a threshold at which the fungicide no longer controls disease.^5^ Resistance mutations can often exist in populations at low frequencies where fungicide efficacy is unaffected, often present as standing genetic variation.^6^ There are numerous examples of fungicide resistance causing field failure in several pathogens and fungicide modes of action. For example, a high frequency of the G143A mutation, associated with quinone outside inhibitor [Q_O_I, Fungicide Resistance Action Committee (FRAC) Group 11]^7^ fungicide resistance, in *Zymoseptoria tritici* resulted in field failure of azoxystrobin in Great Britain.^8^ Similarly, the detection and spread of S524T mutations in *ERG11* have been associated with a loss of demethylation inhibitor (DMI, FRAC Group 3)^7^ fungicide efficacy in *Z. tritici* in Ireland.^9^ By contrast, high frequencies of G460S mutations in *ERG11*, resulting in reduced sensitivity to DMI fungicides, did not cause a loss of DMI fungicide efficacy in comparison with other modes of action for control of *Pyrenopeziza brassicae* in UK field trials.^10^

Some mutations associated with fungicide resistance also result in a fitness penalty in which, in the absence of a fungicide, the resistant isolate is less fit than the sensitive isolate.^3^, ^11^ These fitness penalties may be due to affected enzyme activity from a point mutation or the high energy costs of gene overexpression.^11^ The presence of fitness penalties will aid any resistance management strategies because the frequency of resistance in a population will decrease when no fungicides are applied. The magnitude of the fitness penalty is highly variable and is dependent on the pathogen species and the mutation.^11^ Fitness penalties have been attributed to the lack of DMI resistance development in early blight (caused by *Alternaria alternata*) of potato crops, despite widespread use of difenoconazole for more than 20 years.^12^ By contrast, DMI‐ and Q_O_I‐resistant *Monilinia fructicola* isolates developed through *in vitro* mutagenesis showed little to no observable fitness penalties.^13^ Potential fitness penalties have also been observed in *Leptosphaeria maculans* associated with virulence towards canola (*Brassica napus*) resistance genes. Huang *et al*.^14^ showed that isolates with the virulent allele *avrLm4* produced fewer lesions that were smaller than isolates with the avirulent *AvrLm4* allele. The frequency of *AvrLm4* also increased between leaf lesions collected during autumn and ascospores collected from stubble post‐harvest, likely indicating a fitness penalty associated with virulence. By contrast, no difference in *L. maculans* lesion size was detected between DMI‐resistant and wild‐type isolates when inoculated onto untreated plants, possibly indicating equal fitness in terms of pathogenicity.^15^

High levels of fungicide resistance can have significant economic impacts on farmers in terms of both the cost of the ineffective fungicides and the yield losses due to uncontrolled disease.^16^ It is therefore important to manage fungicide resistance to enable long‐term, effective disease control to maintain maximum yield potential.^17^ Fungicide resistance management strategies have the ultimate aim of either preventing resistance from occurring or maintaining resistance levels below a threshold at which fungicide efficacy is impacted, and the success of these strategies is often dependant on fitness penalties or negative cross‐resistance.^1^ This involves an integrated disease management strategy in which non‐chemical control methods, such as choosing disease‐resistant cultivars and crop rotations, are used where possible.^1^, ^18^ Other chemical strategies may include optimising application timings and doses as well as applying different fungicide modes of action in mixtures or alternations. Another important aspect of resistance management is monitoring for resistance to assess the efficacy of strategies and to determine whether the frequency of resistance below the threshold for field failure.^1^, ^5^

*Leptosphaeria maculans* (cause of blackleg disease, synonym *Plenodomus lingam*) is one of the most significant pathogens affecting canola in Australia, Canada and Europe, with 5–30% yield losses being common.^19^, ^20^, ^21^ Blackleg disease can also be caused by *L. biglobosa* (synonym *P. biglobosus*) but *L. maculans* is the predominant species in Australia and is the focus of this study. The main cause of yield loss in canola from blackleg disease is from crown canker. Crown cankers form at the base of the stem and are caused by infections on cotyledons and up to the fifth‐leaf stage. Protecting canola seedlings and early true leaves from infection is the most effective way of preventing cankers forming later in the season.^22^ Control of blackleg disease involves a combination of cultural practices, host genetic resistance and fungicides. DMI fungicides are used widely across all major canola‐growing regions worldwide, with an increasing reliance on fungicides for disease control.^23^, ^24^, ^25^, ^26^ Fungicide resistance to the DMI chemistries was first detected in Australia in *L. maculans* in 2015 as reduced sensitivity towards the DMI fungicide flutriafol.^27^ The mutations associated with resistance are insertions in the promoter region of the *ERG11* gene, which encodes the target protein for DMI fungicides, resulting in an overexpression of *ERG11*.^28^ Since the first report in 2015, DMI resistance has become widespread across Australia and Europe.^15^, ^29^, ^30^, ^31^ DMI resistance can also be caused by point mutations in the *ERG11* coding region in other species.^4^ However, to date, no coding region mutations have been identified in *L. maculans*,^15^ but in *L. biglobosa* the mutation G476S has been identified as causing resistance in Poland,^32^ meaning there is the potential for target site mutations to occur in *L. maculans*. As such, monitoring strategies should encompass both promoter and target site mutations. New molecular and bioinformatic techniques and tools can detect, monitor and quantify fungicide resistance in *L. maculans*. For example, a deep amplicon sequencing (DAS) assay has also been developed to quantify the frequency of resistance mutations in populations.^15^ Many DMI‐resistant isolates with various *ERG11* promoter insertions have been collected and characterised. Here we use these tools and resources to explore experimentally the dynamics of fungicide resistance in fungal populations and how that could impact management strategies.

## 2 MATERIALS AND METHODS

### 2.1 Preparation of inoculum

Nine isolates (Table 1) were removed from long‐term storage (room temperature with silica dry beads) as cellulose discs and grown onto 10% V8 media amended with ampicillin to prevent bacterial contamination. Isolates were grown at 22 °C with a 12:12 h light/dark photoperiod at all times. To collect pycnidiospores, agar plugs were spread onto fresh 10% V8 media and left to grow for 10 days. These plates were flooded with sterile water and lightly scraped with a scalpel. The spores were filtered through Miracloth and counted using a haemocytometer under a compound microscope. All isolates were diluted to 1 × 10^7^ spores ml^−1^ then recounted to verify the concentration.

**Table 1:** Details of isolates used in experiments in 2021 and 2022

Isolate	Mating type	*ERG11* allele	Fluquinconazole EC_50_ (ng/mL)	Experiment year	Ref.
D3	MAT1‐1	Wild type	50.03	2021, 2022	
D20	MAT1‐2	Wild type	70.40	2021, 2022	
D22	MAT1‐1	Wild type	65.40	2021, 2022	
D25	MAT1‐2	Wild type	51.11	2021, 2022	
18BL151	MAT1‐2	103:ins:275	467.82	2021, 2022	^15^
18BL152	MAT1‐2	103:ins:275	164.90	2021	^15^
20BL196	MAT1‐1	99:ins:237	208.70	2021, 2022	^15^
20BL198	MAT1‐2	132:com:296:16	117.27	2022	^15^
20BL201	MAT1‐1	414:ins:275	83.70	2022	^15^

EC_50_ refers to the dose of fluquinconazole that inbibits 50% of the isolates growth. ERG11 insertion and fluquinconazole sensitivity from Scanlan *et al*.^15^

To create the spore populations used in the experiments the DMI susceptible isolates (D3, D20, D22 and D25) were mixed in equal proportions to create a susceptible master population. Isolates with an *ERG11* insertion (18BL151, 18BL152, 20BL196, 20BL198 and 20BL201) were mixed in equal proportions to create a DMI‐resistant master population, with both master populations at a concentration of 1 × 10^−7^ spores ml^−1^. Four populations with varying frequencies of DMI resistance were developed through serial dilution of the master populations. An aliquot of the resistant master population was diluted with the susceptible master population to generate populations that were made up of 0.01%, 0.1%, 1% and 10% of the DMI‐resistant population. These populations are referred to as Generation 0. All isolate populations were stored in 5‐ml aliquots at 1 × 10^−7^ spores ml^−1^ at −20 °C until use. Fresh isolate populations were made at the beginning of each year.

### 2.2 Glasshouse experiments

Canola (cv Pioneer 43C80) seeds either untreated or treated with fluquinconazole [trade name Jockey Stayer®, 3.34 g of active ingredient (ai) kg^−1^ seed] were sown into 50 × 50 pmm tubes and were watered and fertilised as required throughout the experiments. Each of the three biological replicates were made up of ten plants (technical replicates). Cultivar Pioneer 43C80 was used in these experiments because it has no effective major gene resistance, low levels of quantitative resistance and phenology that allowed suitable flowering times in the controlled environment and short growth height. Canola seedlings were inoculated repeatedly at cotyledon stage, first leaf, second leaf, third leaf and fourth leaf, which occurred at ~7‐day intervals, to mimic repeat infection events in the field. At each timing, all leaves and cotyledons were inoculated by first wounding with tweezers then spraying the plants with spores until run‐off. The spore aliquots were removed from storage, defrosted and diluted to 1 × 10^6^ spores ml^−1^ before inoculation.

Pathogenicity was measured on cotyledons 21 days post the first inoculation by estimating disease severity on each cotyledon lobe on a 1–9 scale, as previously described.^33^, ^34^ At maturity, disease severity was measured on each plant by cutting each plant at the crown and estimating the proportion of the stem infected with disease as Cross‐Section Internal Infection (CSII), as previously described by Marcroft *et al*.^35^ Disease severity data at the cotyledon stage and maturity are presented as the reduction in disease severity from fungicide treatment as a percentage of untreated. The stubble from each replicate was collected into mesh bags and placed on the ground in the field to mature to be used in the DAS assay.

### 2.3 *In planta* fungicide resistance screening

In 2023, 20 fields (Fig. 1 and Table 2) were selected from South Australia to be screened for fungicide resistance.^36^ Canola stubble was collected from the 20 fields, each representing an individual blackleg population, in the Eyre Peninsula region of South Australia, and matured in the field over autumn 2024. The stubble was moistened and primed in a humidity chamber for 24 h in 100% humidity before inoculation. Canola (cv ATR Stingray) seeds either untreated or treated with fluquinconazole (3.34 g ai kg^−1^ seed) were sown into four‐well punnets with two replicate punnets per treatment and randomised within a tray. This cultivar was used in these experiments because it has no effective major gene resistance and for consistency with previous experiments. All plants were grown in a glasshouse at a constant 22 °C and watered as required. The 8‐day old seedlings were inoculated by being placed into plastic tubs with the stubble suspended above and the lid closed.^15^, ^27^ The stubble was kept wet continuously for 2 days to promote ascospore release before being removed. The seedlings were left to grow for a further 10–14 days for disease symptoms to progress. A resistance score (RS) was calculated for each stubble as the ratio of disease on the treated compared with untreated.^15^ A high RS value indicates high levels of resistance in that population. The stubble was then placed back into the field until needed for ascospore liberation.

**Figure 1:**
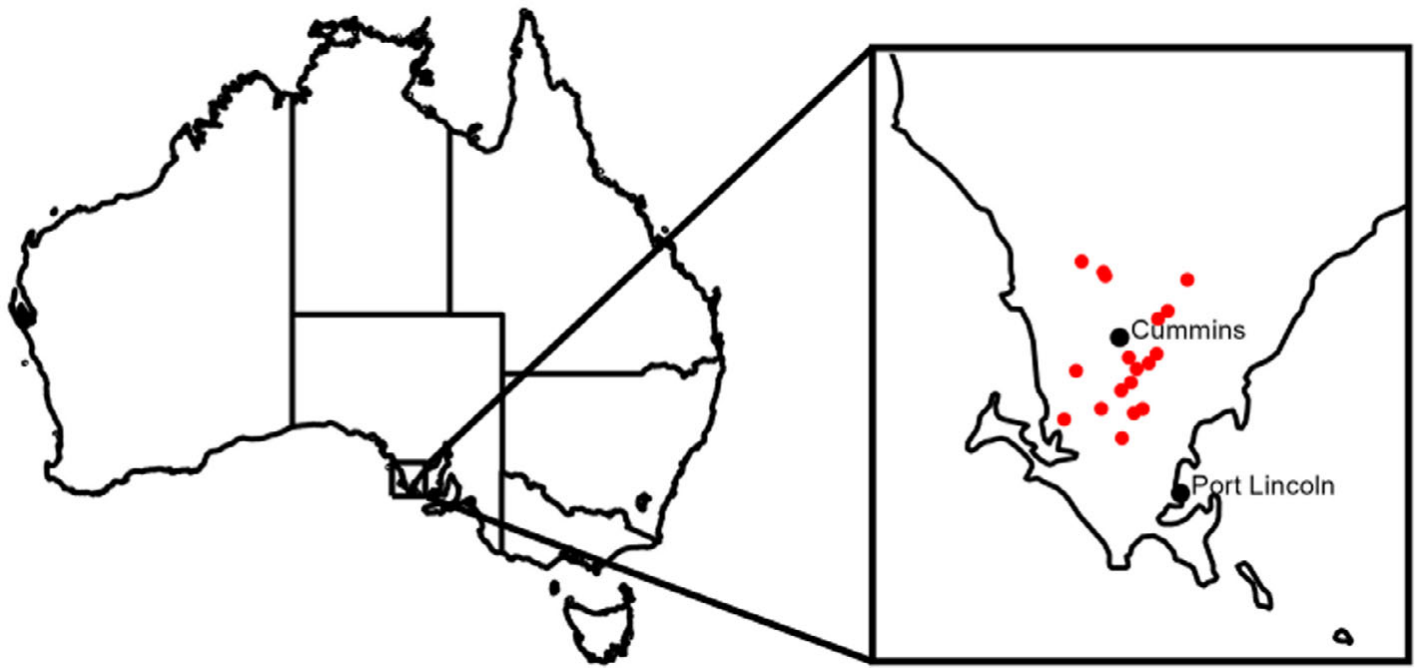
Map of Australia. The inset shows the Eyre Peninsula, South Australia, with field site locations shown in red.

**Table 2:** Locations of the 20 field sites in South Australia and the fungicide resistance data including fluquinconazole resistance score (RS) from the fungicide sensitivity assay, resistance frequency from the deep amplicon sequencing assay and the treatment disease ratios (F/UT and FPA/UT) from the field pots

Site	Locality	Fluquinconazole RS	DMI resistance (%)	F/UT	FPA/UT
1	Wanilla	1.00	4.59	0.59	0.00
2	Wanilla	0.88	30.05	0.26	0.14
3	Coulta	0.63	19.52	1.00	0.00
4	Wanilla	0.94	2.45	0.53	0.07
5	Wanilla	0.40	2.79	0.16	0.00
6	Edillilie	0.50	2.92	0.14	0.00
7	Wangary	0.29	N/A	1.77	0.00
8	Edillilie	0.86	14.75	0.41	0.05
9	Cockaleechie	0.81	2.18	1.36	0.00
10	Edillilie	1.00	2.22	0.36	0.00
11	Warrow	0.13	2.75	0.20	0.00
12	Koppio	0.53	3.00	0.14	0.00
13	Cummins	0.94	24.30	0.67	0.17
14	Cummins	0.73	2.37	0.13	0.00
15	Wangary	1.00	4.65	0.18	0.00
16	Yeelanna	0.80	2.37	0.64	0.00
17	Brimpton Lake	0.64	10.97	0.13	0.00
18	Yeelanna	0.40	3.42	0.53	0.00
19	Karkoo	0.00	1.81	0.00	0.00
20	Cockaleechie	0.85	2.43	0.39	0.00

Fluquinconazole RS, F/UT and FPA/UT are all calculated as the ratio of disease symptoms on the treated compared with untreated plants. Note the demethylation inhibitor (DMI) resistance frequency is not available for site 7 because of poor ascospore release during liberation.

F, fluquinconazole; FPA, fluquinconazole + pydiflumetofen + azoxystrobin; N/A, not determined; UT, untreated.

### 2.4 Field trial using plants cultivated in pots

To test if field failure can occur, canola (cv InVigor® 4520P, rated as moderately resistant–moderately susceptible to blackleg disease^37^) seed was sown into pots filled with Scotts Osmocote Premium Plus Superior Potting Mix placed into 20 fields in South Australia (Fig. 1 and Table 2) where stubble was collected for the *in planta* fungicide resistance screen. Cultivar InVigor®4520P was used because it had suitable phenology for the region, no effective major gene resistance and low levels of quantitative resistance. Seed was sown untreated (UT), treated with fluquinconazole (F) at 3.34 g ai kg^−1^ seed or treated with a mixture of fluquinconazole (DMI, FRAC Group 3), pydiflumetofen [succinate dehydrogenase inhibitor (SDHI), FRAC Group 7]^7^ and azoxystrobin (Q_O_I, FRAC Group 11), referred to as FPA (fluquinconazole + pydiflumetofen + azoxystrobin), rates of 3.34, 0.4 and 0.05 g ai kg^−1^ seed, respectively. The mixture of three fungicides with different modes of action as a seed treatment was included as a baseline measurement, because it is highly unlikely that any isolates are resistant to all three fungicides groups (noting that this is an off‐label use of these fungicides for experimental purposes). Each site contained three pots with four plants per pot and pots were randomised at each site. The canola plants were left to grow in the 2‐year‐old canola stubble until they reached the fourth‐leaf stage and were inoculated by naturally occurring ascospores released from the stubble. After reaching the fourth‐leaf stage, the pots were transferred to a shade house to grow to maturity when disease severity was determined by estimating CSII. All plants were watered twice weekly at seedling stages then more regularly in the shade house as plants matured and fertilised at sowing and approximately eighth‐leaf stage. The disease data obtained were expressed as a ratio of CSII on F and FPA treatments compared with the UT control.

### 2.5 Deep amplicon sequencing

A DAS assay was used to estimate resistance allele frequencies. The mature stubble from both the glasshouse experiments and *in planta* fungicide resistance screen was primed by being kept in a humidity chamber at 100% humidity for 12 h before being placed into a Burkard spore liberator for 2 h. Ascospore populations were collected onto glass microscope slides and stored until further use. The spores were scraped from the slides into 10% V8 liquid media and left for 72 h to germinate. The germinated spores and mycelium were then freeze dried. DNA was extracted from the freeze‐dried material using the Qiagen DNeasy Plant Minikit according to manufacturer's instructions. The ascospore populations collected from the glasshouse stubble are referred to as Generation 1.

The promoter region of the *ERG11* gene was amplified with Takara Ex *Taq* DNA polymerase using the primers pERG11‐F (GGTCTACGAAAAGATTGGTGGCA) and pERG11‐R (CCTTCTTAAACAGGAGTTGCTTGAGG) with polymerase chain reaction (PCR) conditions of 98 °C for 2 min then 35 cycles of 98 °C for 10 s, 62 °C for 30 s and 72 °C for 2 min, then a final extension of 72 °C for 5 min. Amplicons were purified using Qiagen QIAquick® PCR Purification Kit according to the manufacturer's instructions and then sequenced at the Australian Genome Research Facility (Melbourne, Australia) using NovaSeq Control software v.1.2.2.48004 and Real Time Analysis v.4.6.7.

Raw sequencing reads were analysed in a pipeline similar to Scanlan *et al*.^15^ and McCallum *et al*.^38^ Briefly, reads were trimmed, decontaminated and filtered into ‘assembly reads’ (average quality >35 and length >100) and ‘mapping reads’ (average quality >25 and length >40) with *bbduk.sh* in BBMap.^39^ The assembly reads were used as the input to *de novo* assemble into contigs and variation graphs using SPAdes v.3.15.5.^40^ VStrains v.1.1.0^41^ was used to extract and reconstruct haplotypes using the SPAdes outputs and the mapping reads. The resulting haplotypes were compared with a list of known sequences with duplicates removed and novel alleles added to the list.

Allele frequencies were estimated with kallisto v.0.48.0^42^ using a pseudoalignment of the mapping reads and an index of sequences. For the glasshouse experiments, an index of the alleles in Generation 0 was used (Table 1; Supporting Information, Table S1) and for the 20 field ascospore populations the index contained all known alleles (Supporting Information, Table S2). The estimated transcripts per million was converted to allele frequency by dividing by 10 000.

### 2.6 Statistical analyses

All statistical analyses were conducted in R v.4.3.0.^43^ Visualisations were conducted using the ggplot2 v.3.4.2 package.^44^ Disease severity data from the glasshouse experiments were analysed using analysis of variance, with the *aov()* function in the *stats* package,^43^ and Fisher's Least Significant Difference test, with the *LSD.test()* function in the *agricolae* ^45^ package, with the disease scores from ten plants averaged from each of the three replicates in each year. Student's *t*‐test was used to determine significant changes in resistance frequency with the *t.test()* function in the *stats* ^43^ package. All correlations were determined with the *cor.test()* function in the *stats* ^43^ package using Pearson's product correlation coefficient.

## 3 RESULTS

### 3.1 Determining the frequency of fungicide resistance in *L. maculans* populations needed to render the fungicide ineffective

Canola plants were inoculated throughout the seedling stage with spore populations with different DMI resistance frequencies and were scored for disease at the cotyledon stage and at the crown at maturity. This allowed the frequency of resistance required to overcome the fungicide to be determined. At the cotyledon stage, the frequency of resistance at Generation 0 had a significant effect on the reduction in disease severity due to the fungicide (*P* < 0.001). The fluquinconazole seed treatment reduced disease by approximately 50–60% in 2021 and 80–90% in 2022 for 0–1% resistance frequencies. At 10% and 100% resistance frequency, the efficacy of the fungicide decreased and only reduced disease by 5–20% in 2021 and 40–50% in 2022 (Fig. 2). This indicated that in both years >1% fungicide resistance was needed before visual differences in disease symptoms would occur due to the loss of efficacy of the fungicide. For disease severity measured at maturity, the frequency of resistance at Generation 0 also had a significant effect on the reduction in disease severity due to the fungicide (*P* < 0.001). The fungicide treatment resulted in a 30–50% reduction in CSII for 0–1% resistance frequency in 2021 and a 50–60% reduction for 0–0.1% resistance frequency in 2022 (Fig. 3). The frequency at which fungicide efficacy significantly dropped is different in each year. In 2021, at least 10% resistance frequency was needed at Generation 0 to significantly reduce fungicide efficacy, whereas in 2022 this occurred at 1%. Therefore, for CSII, between 1% and 10% fungicide resistance was needed in a population for it to overcome a fungicide seed treatment.

**Figure 2:**
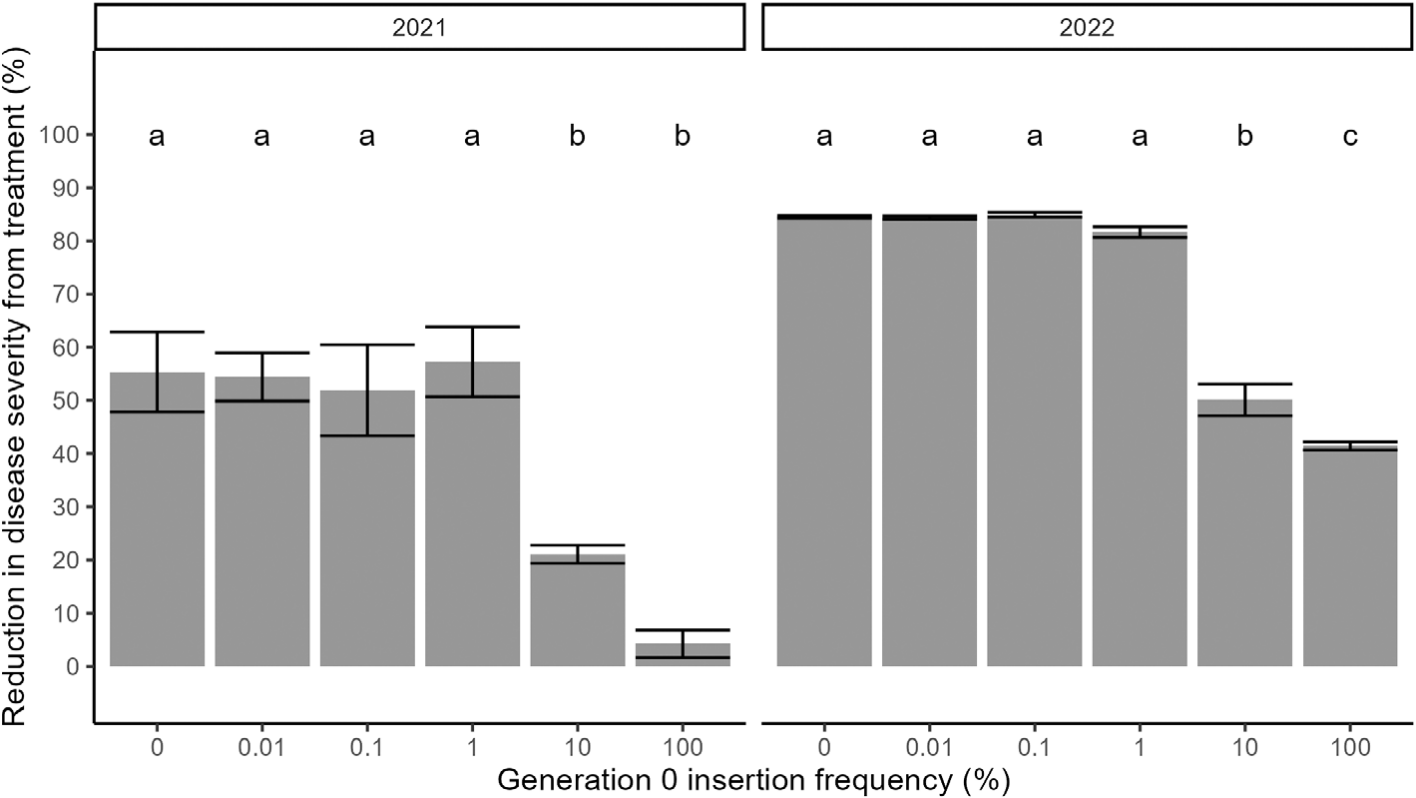
Reduction in cotyledon disease severity from the fluquinconazole seed treatment 21 days post inoculation for each *Leptosphaeria maculans* population in 2021 and 2022. Different letters in each year indicate statistically significant results (*P* < 0.05) based on Fisher's Least Significant Difference and three replicates of ten plants per replicate. Error bars indicate standard error.

**Figure 3:**
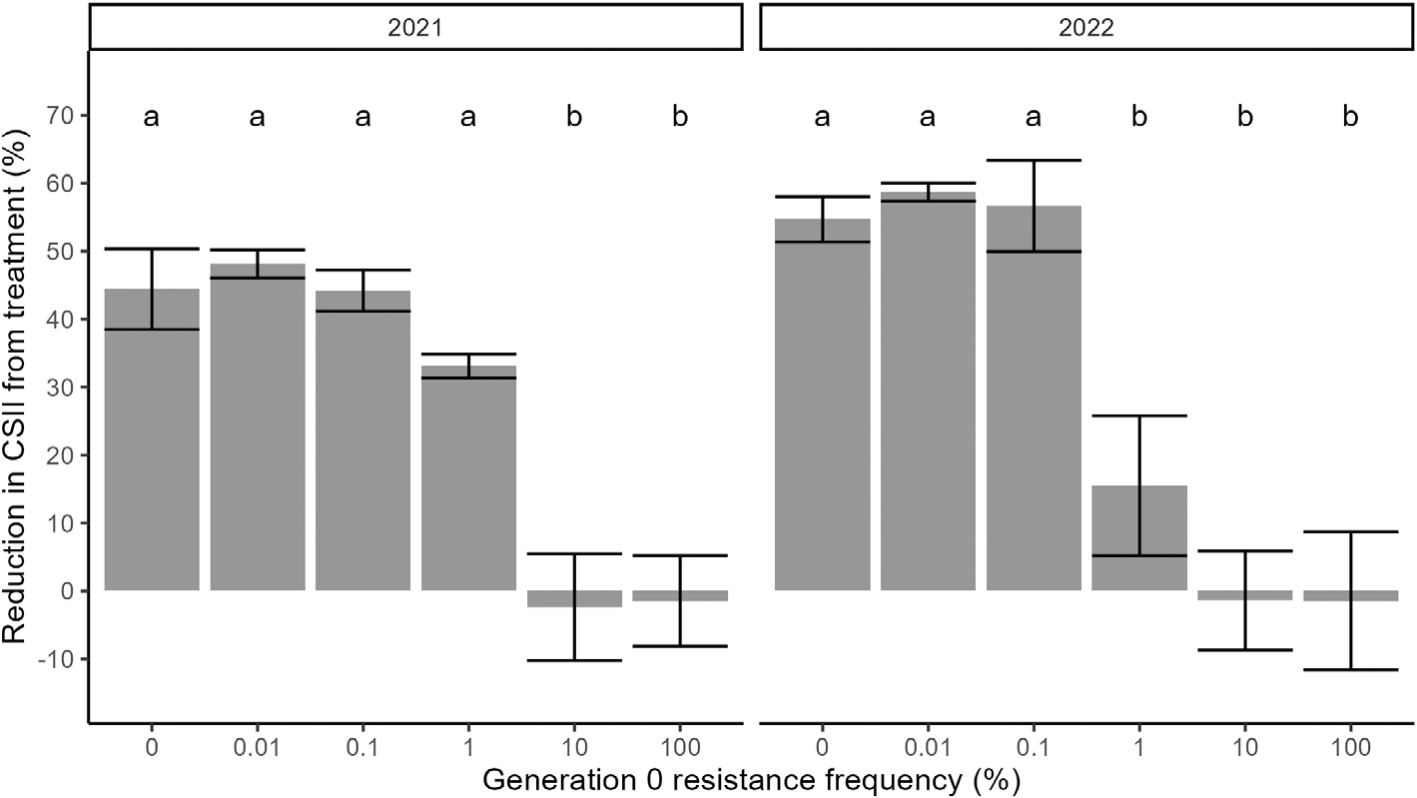
Reduction in Cross‐Section Internal Infection (CSII) disease severity from the fluquinconazole seed treatment 21 days post inoculation for each *Leptosphaeria maculans* population in 2021 and 2022. Different letters in each year indicate statistically significant results (*P* < 0.05) based on Fisher's Least Significant Difference and three replicates of ten plants per replicate. Error bars indicate standard error.

### 3.2 Impact of selection on fungicide resistance frequencies

Ascospore populations (Generation 1) were collected from the mature canola stubble from the previous experiments, and the DAS assay was used to estimate the resistance frequency of each population. This allowed calculation of the change in frequency of resistance following 1 year of selection and whether the level of resistance would increase or decrease in the presence or absence of the DMI fungicide. When the resistance frequencies of Generation 0 populations were 0%, 0.01% or 0.1%, the resistance frequency of Generation 1 did not significantly differ from the initial Generation 0 for both the untreated and fungicide treatment (Fig. 4). At 1% resistance frequency of Generation 0, the fungicide treatment significantly increased the resistance frequency to 15.4% in Generation 1 and without the fungicide, it significantly decreased by 0.51%. At 10% resistance frequency at Generation 0, the fungicide treatment increased the resistance frequency by 34.4% in Generation 1 but the untreated did not significantly differ from Generation 0.

**Figure 4:**
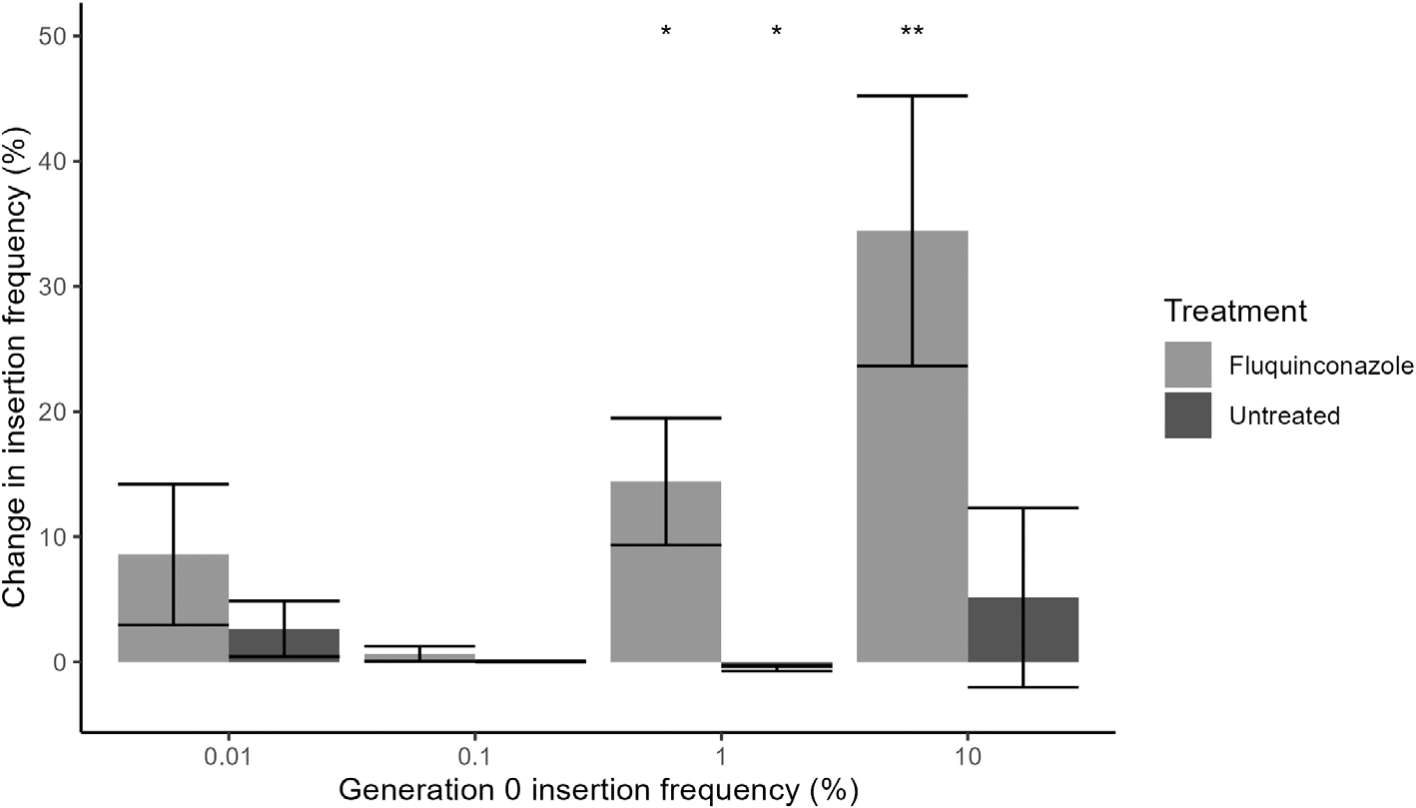
Change in resistance frequency for each population either treated with fluquinconazole or untreated. The results for 2021 and 2022 were combined. Change in resistance frequency calculated as Frequency at Generation 1 − Frequency at Generation 0. Columns marked by a symbol are significantly different from zero (*P < 0.1, **P < 0.05) based on Student's *t*‐test and three replicates of ten plants per replicate. Error bars indicate standard error.

### 3.3 Analysis of fungicide sensitivity in *L. maculans* populations

The 20 *L. maculans* populations were assessed for fungicide sensitivity using the *in planta* assay. An RS value was calculated for each population with one population having an RS of 0 and the RS values of the other 19 populations ranging from 0.13 to 1.0, with an overall mean of 0.67 (Table 2). The 20 populations were allowed to undergo sexual reproduction, the resulting ascospores liberated and the frequency of resistance determined in the next generation. The DMI resistance frequency varied from 1.8% to 30.1% with a mean of 7.3% (Table 2). Population 7 failed to liberate sufficient ascospores, so the DMI resistance frequency was not determined. There was a poor correlation between resistance frequency and RS (*r* = 0.275, *P* = 0.255). Four resistant alleles were present at a frequency >5% in at least one population. These were −87:ins:187 (8.82% at site 17), −90:ins:153 (10.92% at site 3), −116:com:296:16 (26.91% at site 2) and −414:ins:275 (21.97% at site 13 and 12.44% at site 8). In the five populations with >5% resistance, there was one allele that made up the majority of the total resistance frequency (Supporting Information, Data S1).

### 3.4 Correlation with failure in the field

Canola was sown into pots placed into 20 fields located in South Australia, where canola stubble was collected previously, and grown to maturity when CSII was measured. The F/UT ratio varied from 0.00 at site 19 to 1.77 at site 7, with a mean of 0.42, while the FPA/UT ratio was 0.00 at 16 of the sites with a maximum of 0.17 at site 13 (Table 2). Higher ratios indicate higher levels of disease on the fungicide treatments relative to the untreated control and demonstrate a level of field failure. A ratio >1 indicates higher disease in the treatment compared with the untreated. As illustrated in Fig. 5, there was a low correlation between F/UT ratio and resistance frequency (*r* = 0.168, *P* = 0.491) or RS (*r* = 0.0592, *P* = 0.804).

**Figure 5:**
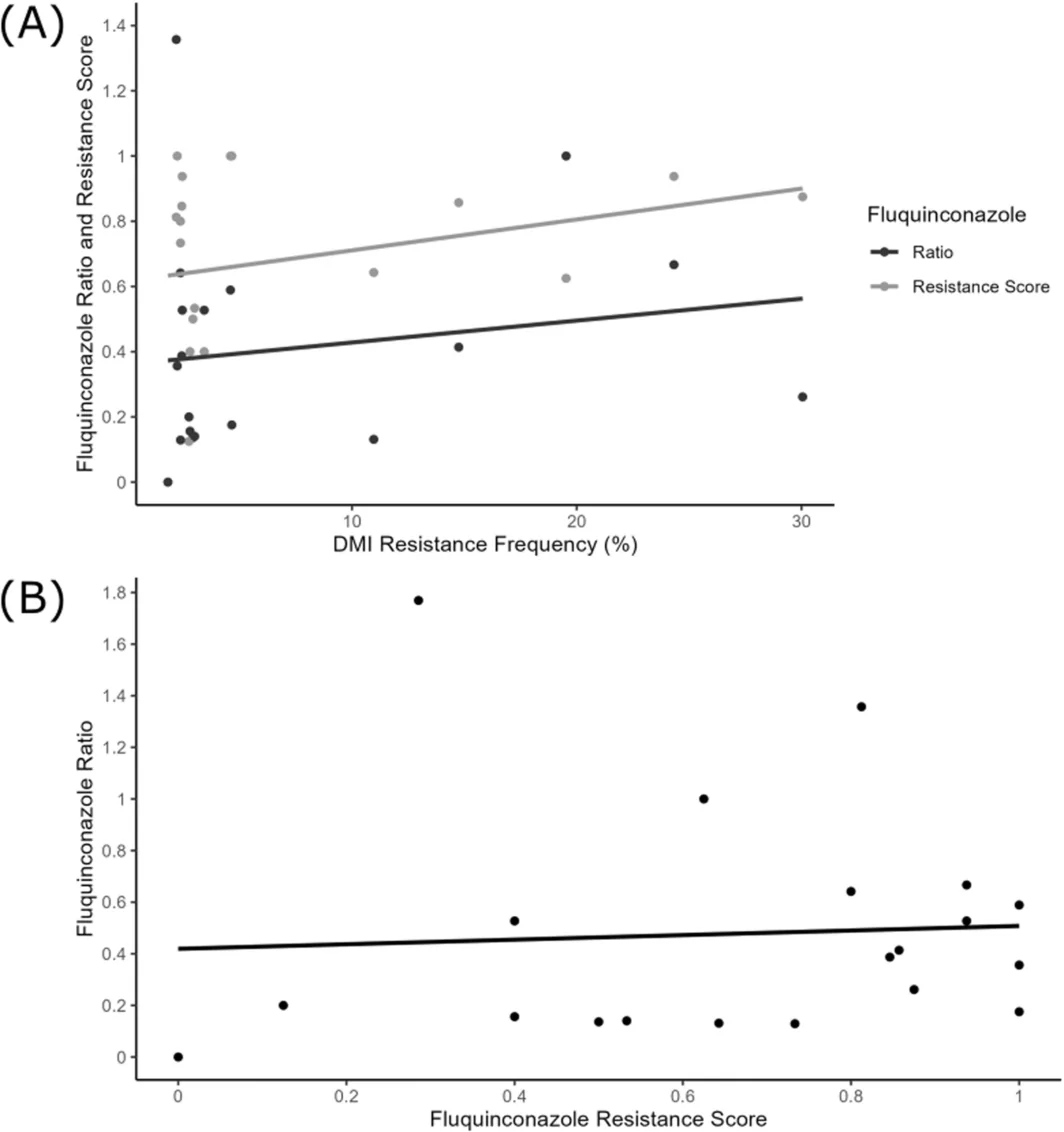
(A) Relationship between population demethylation inhibitor (DMI) resistance frequency and fluquinconazole ratio (F/UT, *R* ^2^ = 0.029, *P* = 0.491) and fluquinconazole resistance score (RS, *R* ^2^ = 0.076, *P* = 0.255). (B) Correlation between fluquinconazole ratio (F/UT) and resistance score (RS, *R* ^2^ = 0.052, *P* = 0.804). Data from 20 *Leptosphaeria maculans* populations in South Australia. F, fluquinconazole; UT, untreated.

## 4 DISCUSSION

The increase in fungicide resistance in many pathogens worldwide has serious implications for disease control. Fungicide resistance becomes an issue when it reaches a level at which fungicides are no longer providing disease control. For field failure to occur the resistance factor of the isolates needs to be high enough for the isolates to be unaffected by the application rates of the fungicide and the frequency of these resistant isolates needs to be high enough in the population for them to cause sufficient disease.^3^ Other trends such as incorrect timing, poor application, very high disease pressure or growing susceptible varieties can also contribute to the failure of a fungicide to control disease.^3^, ^46^, ^47^ It is important to identify and quantify the frequency of resistance required for field failure to occur to ensure that monitoring can detect resistance below this level. From the glasshouse experiments, it was evident that field failure of fluquinconazole occurred at 1% DMI resistance in Generation 0 in 2022 and 10% resistance in 2021. Interestingly in 2022 at 1% resistance, there was a significant reduction in disease symptoms on the cotyledons but no reduction in CSII at maturity. Therefore, at this level of resistance it would appear that no fungicide resistance is present owing to the lack of lesion development at the seedling stage, but the resistance is high enough that crown cankers can still occur later in the season to cause yield losses. Quantifying fungicide resistance in a population is important because fungicide resistance management strategies may need to be adapted depending on the level of resistance in the population, as the initial resistance frequency can impact the efficacy of different strategies.^48^

Of the 19 field populations with known DMI resistance frequencies, none are below 1%, 14 are between 1% and 5% and five are above 5% resistance. There is, however, a poor correlation between disease severity on the DMI treatment and the frequency of DMI resistance in the populations (*r* = 0.168, *P* = 0.491) (Fig. 5). For example, site 16 had a F/UT ratio of 0.64 and a DMI resistance of 2.37%, whereas site 2 had a ratio of 0.26 and a resistance frequency of 30.1%. Sites 7 and 9 had an F/UT ratio >1, indicating there was higher disease in the fluquinconazole treatment compared with the untreated control. The lack of correlation between disease severity and resistance frequency infers that although field failure does occur, there may be other confounding variables impacting field failure such as timing of infection or disease pressure. Although in the glasshouse experiments at most 10% resistance is needed to reduce the fungicide efficacy completely, this has not translated exactly into the field experiments. It may be that the threshold for field failure shifts depending on other factors and for early, very high disease pressure scenarios, a lower level of resistance is needed to overcome the fungicide compared with a season with later infections at lower disease pressure where a higher level of resistance may be needed to overcome the fungicide. Resistance to the DMI fungicide prothioconazole has been shown to cause field failure in the control of septoria tritici blotch (caused by *Zymoseptoria tritici*) in Europe. Field trials located across Europe showed varying levels of control, with some sites showing a lack of control and lower yields compared with the more effective fungicide mefentrifluconazole.^49^

The frequency of resistance can also substantially increase following just 1 year of selection. For example, the 1% resistance in Generation 0 increases to >15% in Generation 1 with just a single fungicide application. This has also been shown in the field, with applications of DMI fungicides resulting in increases in resistance mutations in *Z. tritici*.^50^ In the field, resistance could potentially increase in a single season from a level at which it is undetectable to a level at which field failure is likely to occur when only DMI fungicides are used. This also highlights the value of diagnostic tools that are sensitive enough to detect very low frequencies of resistance alleles. For a conventional isolate collection, a sample size from each field of at least 300 isolates is needed to detect rare alleles at a frequency of 1%, with 95% confidence.^51^ This means it becomes logistically impossible to screen a large number of fields and shows that assays, such as the DAS assay and *in planta* assay, that screen tens of thousands of spores are an important aspect of monitoring resistance.

There is limited evidence for a strong fitness penalty to strains carrying mutations in the *ERG11* gene in the glasshouse experiments of this study. DMI resistance in *L. maculans* is caused by insertions in the promoter region of *ERG11* resulting in overexpression of the gene.^28^ Overexpression of this gene in other fungi can result in a high energy cost and resource allocation that can lead to a fitness penalty.^11^, ^52^, ^53^ If a strong fitness penalty was present for DMI resistance, then the frequency of resistance would decrease from Generation 0 to Generation 1 in the absence of any selection pressure. The frequency of resistance does decrease slightly for two no‐fungicide treatments, from 1% in Generation 0 to 0.48% in Generation 1 (*P* = 0.064), but does not significantly decrease for any other treatments. Therefore, there may be a slight fitness penalty that can cause the level of resistance to decrease from high levels, but it is likely not strong enough to completely remove the resistance from the population, and thus resistance is likely to persist at low levels even in the absence of fungicide use. High frequencies of insertions of DNA fragments into the promoter of *ERG11* have been detected in Europe and Australia, with some European populations varying between 85% and 100% and up to 32% in Australia.^15^, ^30^ This high level of resistance may be due to the lack of a fitness penalty or sustained use of DMI fungicides over a long period. DMI resistance is also caused by a variety of insertions that may have different fitness levels because of different *ERG11* expression levels^28^ and the change in resistance frequency may depend on the resistant genotypes in the population. For example, in Europe, *Z. tritici* isolates with the mutation C‐H152R, which confers high‐level resistance to SDHI fungicides, are present at a low frequency compared with other mutations such as C‐T79N and C‐N86S, which confer low–moderate resistance but are present at high frequencies. This infers a fitness penalty in C‐H152R isolates that keep frequencies low, but no fitness penalty in C‐T79N or C‐N86S that allows the frequency of these mutations to remain high in populations.^54^ There are limited examples of fitness penalties caused by DMI resistance in plant pathogens, with the majority of mutations having no effect on growth or pathogenicity.^11^ Some examples of potential fitness penalties include the increase in prochloraz resistance in *Oculimacula yallundae* and *Oculimacula acuformis*, the causal pathogens of eyespot disease in cereals, over many years, followed by the replacement of prochloraz with alternative fungicides resulting in a decrease in prochloraz resistance in *O. yallundae* but no change in *O. acuformis*, and an increase in the relative abundance of *O. yallundae* compared with *O. acuformis*.^55^ Prochloraz‐resistant isolates of *Oculimacula* spp. have also been found to be either equally or less competitive than sensitive isolates, highlighting a likely fitness penalty for prochloraz resistance.^56^, ^57^ Similarly, the frequency of *Phakopsora pachyrhizi ERG11* mutants decreased when in competition with wild‐type isolates in the absence of a fungicide, also indicating the presence of a fitness penalty.^58^ The success of fungicide resistance management strategies can be dependent on the presence of fitness penalties, or negative cross‐resistance^1^, so it is important to determine whether these trends occur.

## 5 CONCLUSION

Management of fungicide resistance is an important issue facing farmers worldwide. This study has shown that DMI resistance in *L. maculans* populations is widespread and at high enough levels that failure to control blackleg disease in the field could occur in Australian fields. The low threshold of strains within the population for field failure means that any routine monitoring will need to detect accurately resistance below that level, and to ensure ample warning for farmers to change their practices before field failure occurs. No strong fitness penalty was detected associated with mutations in the *ERG11* gene, and it is likely that resistance will endure in the absence of a fungicide. Farmers who rely on sole DMI fungicide applications to control blackleg disease in canola may find such a strategy ineffective in the level of control it provides. A resistance management strategy should be employed to use tactics including switching or mixing with alternative fungicides (such as SDHI or Q_O_I), isolating canola crops from previous years canola stubble to reduce disease pressure or growing cultivars with blackleg resistance. It is also important to monitor resistance to fungicides where resistance has evolved to determine the efficacy of management strategies, as well as alternative fungicides with no detected resistance, such is the case for SDHI and Q_O_I resistance in *L. maculans*,^26^, ^59^ to ensure resistance has not evolved.

## FUNDING INFORMATION

The authors acknowledge the Australian Research Council (IM230100025), Grains Research and Development Corporation (UOM2304‐004RSX), Syngenta and Marcroft Grains Pathology for providing funding for this research.

## CONFLICT OF INTEREST

The authors declare no conflict of interest.

## Supporting information


**Data S1.** Supporting Information.

## Data Availability

The data that support the findings of this study are available from the corresponding author upon reasonable request.
